# The Effect of Unihemispheric Concurrent Dual-Site Transcranial Direct Current Stimulation of Primary Motor and Dorsolateral Prefrontal Cortices on Motor Function in Patients With Sub-Acute Stroke

**DOI:** 10.3389/fnhum.2018.00441

**Published:** 2018-10-31

**Authors:** Sahar Toluee Achacheluee, Leila Rahnama, Noureddin Karimi, Iraj Abdollahi, Syed Asadullah Arslan, Shapour Jaberzadeh

**Affiliations:** ^1^Department of Physiotherapy, University of Social Welfare and Rehabilitation Sciences, Tehran, Iran; ^2^Pediatric Neurorehabilitation Research Center, University of Social Welfare and Rehabilitation Sciences, Tehran, Iran; ^3^Department of Physiotherapy, School of Rehabilitation, International Campus, Tehran University of Medical Sciences, Tehran, Iran; ^4^Non-invasive Brain Stimulation and Neuroplasticity Laboratory, Department of Physiotherapy, School of Primary and Allied Health Care, Faculty of Medicine, Monash University, Melbourne, VIC, Australia

**Keywords:** tDCS, stroke rehabilitation, upper extremity, motor skills, motor cortex, stroke

## Abstract

It is believed that unihemispheric concurrent dual-site transcranial direct current stimulation (tDCS_UHCDS_) of the primary motor cortex (M1) and the dorsolateral prefrontal cortex (DLPFC) causes an increase in motor cortex excitability. However, the clinical effect of this type of stimulation on patients with neurological conditions is not yet known. The aim of the present study was to assess the effect of anodal-tDCS_UHCDS_ (a-tDCS_UHCDS_) on upper limb motor function in subacute stroke patients. Fifteen patients participated in this sham-controlled crossover study. The main outcome measures were the reaction time (RT) to visual stimuli, completion time of a nine-pin pegboard (9-PPB), and the scores from the Fugl–Meyer assessment (FMA) for the upper limb of the involved side before and after three brain stimulation conditions. For a-tDCS_UHCDS_, the anodal electrodes were placed on the M1 and the DLPFC, while for a-tDCS, the anodal electrode was placed on the M1. For the sham stimulation, the tDCS was turned off after 30 s. For brain stimulation, the selected current was 1 mA for 20 min. After a-tDCS_UHCDS_, there was a significant reduction in the RT and completion time of the 9-PPB compared with the times after a-tDCS and the sham stimulation: *p* = 0.013 and *p* = 0.022, respectively). However, there was no significant difference in the FMA scores after the three types of stimulations (*p* = 0.085). Compared with a-tDCS, a-tDCS_UHCDS_ temporarily improved the RT and dexterity of the involved hand in subacute stroke patients.

**Clinical Trial Registration:** Iranian Registry of Clinical Trials (IRCT), identifier IRCT2015012520787N1.

## Introduction

Stroke is considered to be the second-leading cause of mortality and the major reason for disability in adults all over the world (Kuklina et al., 2012). Stroke causes motor, sensory, and cognitive deficits (Mayo et al., 1999). Upper limb weakness, which is seen in more than 40% of stroke patients, is one of the most important symptoms in both acute and chronic conditions (Parker et al., 1986). After a stroke, the recovery of motor function is very important because it enables stroke survivors to independently perform activities of daily living (Harris and Eng, 2007). Recently, a number of studies have investigated the impact of non-invasive brain stimulation (NIBS) on the enhancement of neuroplasticity and the recovery of symptoms caused by brain lesions (Hummel and Cohen, 2006; Summers et al., 2007; Kim et al., 2016; Satow et al., 2016; Andrade et al., 2017). Transcranial direct current stimulation (tDCS) is a NIBS technique that uses low-intensity direct current to modulate the excitability of neurons in different cortical sections and deep areas of the brain (Nitsche and Paulus, 2000; Liebetanz et al., 2002; Kim et al., 2012). Previous studies have suggested that the application of tDCS to the primary motor cortex (M1) can improve upper limb motor function. For example, anodal tDCS (a-tDCS) of the M1 improved the motor function of the hand in both healthy individuals and stroke patients (Bastani and Jaberzadeh, 2012; Butler et al., 2013; Lee and Lee, 2015; Fleming et al., 2017). A-tDCS may also lead to a decrease in reaction time (RT) in patients performing different motor tasks (Hummel and Cohen, 2005; Hummel et al., 2005, 2006; Boggio et al., 2006). It has been observed that separate a-tDCS of the M1 and the dorsolateral prefrontal cortex (DLPFC) increases the excitability of the motor cortex in healthy individuals (Vaseghi et al., 2015a). In addition, the simultaneous stimulation of the M1 of both hemispheres (anodal current over M1 and cathodal current over the contralateral M1) had a greater improvement on motor learning compared with a sham simulation or a unilateral a-tDCS of the M1 (Karok and Witney, 2013; Di Lazzaro et al., 2014). Stagg et al. (2013) reported that a-tDCS of the DLPFC increased the blood flow between the sensorimotor cortex and the DLPFC. Vaseghi et al. (2015b) compared a single-site stimulation of the M1 or DLPFC with the simultaneous unilateral stimulation of M1 and DLPFC using a new protocol called unihemispheric concurrent dual-site a-tDCS (a-tDCS_UHCDS_); they found that a-tDCS_UHCDS_ significantly increases the M1 corticospinal excitability (CSE) in healthy individuals. However, in spite of the significance of this finding in the enhancement of CSE, the clinical and functional implications of a-tDCS_UHCDS_ have not yet been studied. To the best of the authors’ knowledge, a study of the effects of this novel tDCS approach on the motor function of stroke patients has not been conducted.

In the current research, we aimed to compare the effects of conventional single-site stimulation of M1 with a-tDCS_UHCDS_ on upper limb motor function. We hypothesized that a-tDCS_UHCDS_ of the M1–DLPFC decreases the RT and completion time for a nine-pin pegboard (9-PPB) and increases motor function as evidenced by the Fugl–Meyer assessment (FMA) in subacute stroke patients.

## Materials and Methods

### Design

This study had a sham-controlled crossover design. The current study was registered as a clinical trial study in the Iranian Registry of Clinical Trials^1^ with the registration number: IRCT2015012520787N1. All experimental procedures were approved by the Human Ethics Committee of the University of Social Welfare and Rehabilitation Science, Tehran, Iran. This study followed the CONSORT checklist, which is included as a Supplementary File. All participants read and signed a written informed consent form before taking part in the study.

### Participants

**FIGURE 1:**
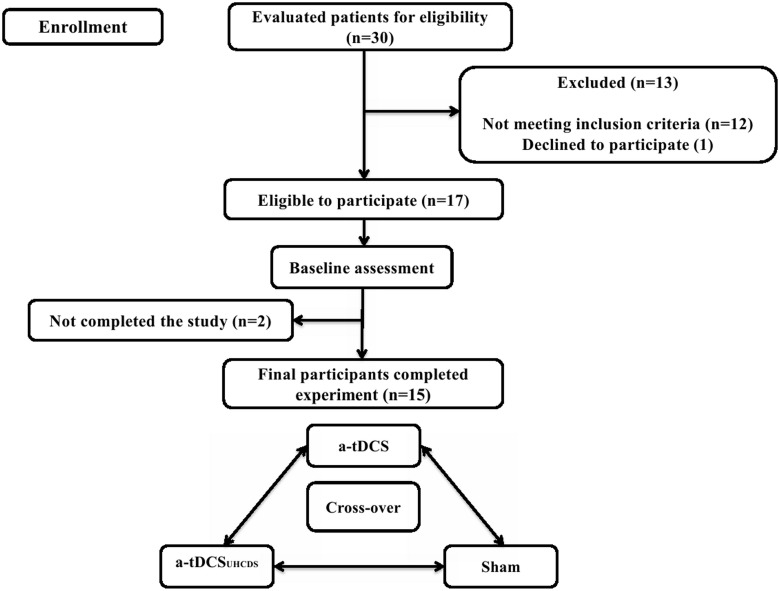
The study procedure flow chart.

Fifteen subacute stroke patients with subcortical lesions voluntarily participated in this study; there were six females and nine males with the average age of 66.17 ± 6.36 years and 63.33 ± 7.14 years, respectively. The participants were selected from a pool of patients undergoing rehabilitation at regional clinics and hospitals. All participants sustained ischemic stroke diagnosed by a neurologist using magnetic resonance imaging. Patients were included in the study if it was their first stroke, they were 40–80 years old, and they had a Brunnstrom score of 3 (marked spasticity, but voluntary synergistic finger movement could be observed). Patients were excluded from the study if they had any other neurological disease except stroke, they had a metal implant in the brain, they had musculoskeletal disorders that affected the upper limbs, they had aphasia, they were taking neuropsychiatric drugs, such as benzodiazepines or antidepressants, and they had scored less than 25 in the Mini-Mental State Examination (MMSE). Table 1 shows the demographic characteristics of the patients and Figure 1 shows the trial procedure flow.

**Table 1:** Patients demographic characteristics.

Patient no.	Age (year)	Time aafter stroke (month)	Lesion site (ischemic site)	Dominant hand	MMSE	Brunnstrom
**1**	68	15	R putamen	R	25	4
**2**	53	8	L pontine	R	27	4
**3**	76	8	R corona radiata	R	29	4
**4**	62	9	L putamen thalamus	R	26	3
**5**	74	20	R internal capsule	R	28	3
**6**	69	12	L putamen	L	28	3
**7**	71	10	R putamen	R	29	3
**8**	55	16	R corona radiata	R	27	4
**9**	63	24	R corona radiata	R	28	4
**10**	61	19	L basal ganglia	L	25	4
**11**	62	20	L putamen	L	29	4
**12**	70	22	R internal capsule	R	25	3
**13**	60	17	R basal ganglia	R	29	4
**14**	65	13	R putamen	R	28	3
**15**	58	11	L corona radiata	L	28	4


*R, right; L, left.*

### Transcranial Direct Current Stimulation

**FIGURE 2:**
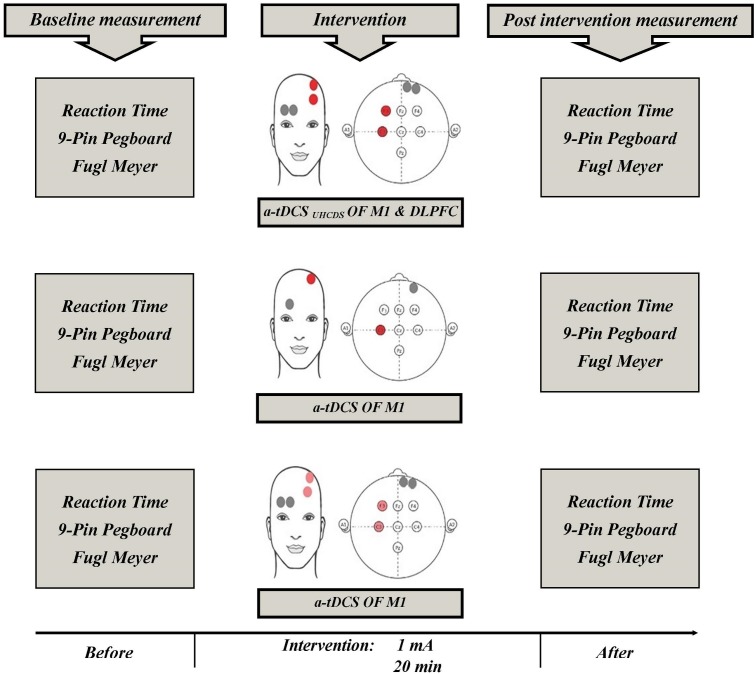
Schematic representation of the experimental protocol with measures taken before and after a-tDCS. a-tDCS, anodal-tDCS; a-tDCS_UHCDS_, anodal unihemispheric concurrent dual-site transcranial direct current stimulation; M1, primary motor cortex; DLPFC, dorsolateral prefrontal cortex.

Three experimental conditions (stimulation type) included a-tDCS_UHCDS_, conventional single-site a-tDCS, and sham a-tDCS. Each participant randomly received all three types of stimulation. A-tDCS_UHCDS_ was applied using two active electrodes (2 cm × 3 cm) placed over the M1 and DLPFC of involved hemisphere and two reference electrodes (2 cm × 6 cm) placed over the contralateral supraorbital area. For the conventional single-site a-tDCS, one active electrode (2 cm × 3 cm) was located over M1 of involved hemisphere and a reference electrode (2 cm × 6 cm) was placed over the contralateral supraorbital area. For the sham stimulation, the electrodes were placed in the same positions used for the stimulation of M1 or M1–DLPFC, but the device was turned off after 30 s of stimulation. The a-tDCS devices were set to deliver 1 mA direct current for 20 min. The current density under the active electrode was 0.016 mA/cm^2^, which was comparable with the current density used in previous studies (Furubayashi et al., 2008; Kwon et al., 2008). Small active electrodes were used in all conditions to increase the focality of the stimulation over the target areas (Nitsche et al., 2007; Kwon et al., 2008; Bastani and Jaberzadeh, 2013). The stimulation sites were based on the international 10–20 electroencephalography standard (Figure 2). Depending on the side of the pathology, active electrodes were located over the right or left M1 (C3 or C4) and right or left DLPFC (F3 or F4). The wash-out period between the different experimental conditions was 72 h (Fujimoto et al., 2016).

### Outcome Measures

#### Reaction Time

Deary–Liewald reaction time software (RT, version 3.10, Centre for Cognitive Ageing and Cognitive Epidemiology, University of Edinburgh, Scotland) was used to assess the hand RT to visual stimuli (Deary et al., 2011). At the start of the test, a number of colored stimuli appeared at random intervals on a laptop screen, which was placed in front of the participant. The participant was asked to press the red slash key on the keyboard as quickly as possible after seeing the stimuli. Participants were in a comfortable position while performing the test; the table height was adjusted such that the person could see the screen and had easy access to the keyboard. Before beginning the study protocol, all participants performed a test trial involving eight stimuli to become familiar with the test. During the study, the program was set to display 20 stimuli. A reduced RT in response to the visual stimuli indicated an improvement in performance. The software has been shown to have good reliability (Deary et al., 2011).

#### Nine-Pin Pegboard Test

The 9-PPB test was developed to investigate the hand dexterity in stroke patients (Oxford Grice et al., 2003). The 9-PPB consisted of two rectangular boards that were attached; one board had nine holes in three rows and other board, which was located above the first, had nine holes with a peg in each holes. The 9-PPB was placed in front of each participant who was instructed to pick up each peg from the top board, one by one, as quickly as possible and place it in a hole in the other board. The total time taken to complete the task was determined using a chronometer and was recorded as the test score for each individual (Croarkin et al., 2004). The chronometer accuracy was 0.01 s. The 9-PPB test has excellent test–retest reliability in acute stroke patients (Heller et al., 1987).

#### Fugl–Meyer Assessment

The FMA was used to assess upper limb motor function in stroke patients (Fugl-Meyer et al., 1974; Nitsche and Paulus, 2000). The upper limb section of the assessment contained 33 items and each item had the following score: 0 = unable to move the limb; 1 = partially able to move the limb; and 2 = fully able to move the limb. The total scores for the upper limb section ranged from 0 to 66. Previous studies have reported excellent inter-rater and intra-rater reliability and good construct validity for the FMA (Gladstone et al., 2002).

### Procedure

Figure 2 shows the experimental setup of the study. Participants were asked to sit on an adjustable chair. Participants became familiar with the testing procedures and the outcome measures, which included the FMA, 9-PPB, and RT. Just before and immediately after each experimental intervention, which were given 72 h apart, the outcome measures were assessed and the participants’ scores were measured. Other factors, such as attention, fatigue, and discomfort, were also assessed using a 7-grade Visual Analog Scale (VAS) to evaluate any adverse or side effects of the tDCS. The participants were asked to show the intensity of their fatigue, attention and discomfort on the 7 cm VAS just before and immediately after the experimental interventions. The scoring methods for the scales were as follows: 1 = no concentration and 7 = maximum concentration; 1 = no fatigue and 7 = maximum fatigue; and 1 = no discomfort and 7 = maximum discomfort, respectively. The VAS scoring used in this process has been reported to have excellent reliability (Aitken, 1969; Scherder and Bouma, 2000).

### Data Analysis

Data were analyzed using SPSS version 21 (IBM Corp., Armonk, NY, United States). The normal distribution of the data was assessed using the Shapiro–Wilk test. The effects of the different stimulation conditions on the outcome measures, which included RT, 9-PPB, and FMA, were evaluated using the repeated measures analysis of variance (ANOVA). A comparison of the baseline measurements before each stimulation condition was conducted using the one-way ANOVA. The Wilcoxon test was used to compare the psychological data before and after each stimulation condition. The level of significance was set at *p* < 0.05.

## Results

The analysis showed that the distribution of the psychological data was not normal. However, the RT, 9-PPB, and FMA data were distributed normally.

### Psychological Data

An analysis of the psychological data revealed that there was no significant difference between the pre- and post-procedure attention, fatigue, and discomfort of the participants. No participant reported a headache or any other adverse effect after receiving the tDCS; however, two participants had the feeling of burning at the point of the active electrode at the start of the stimulation, which was resolved after 1 min. Table 2 shows patients’ perceived VAS scores in centimeter for the assessed psychological variables including fatigue, attention, and discomfort just before and immediately after each experimental condition.

**Table 2:** Visual Analog Scale Scores for fatigue, attention, and discomfort measurements before and after each intervention.

	Discomfort (Mansur et al., 2005)		Attention (Mansur et al., 2005)		Fatigue (Mansur et al., 2005)	

	After	Before	After	Before	After	Before
Sham	1.60 ± 0.91	1.73 ± 1.03	6.87 ± 0.35	6.80 ± 0.41	1.00	1.13 ± 0.35
	*p* = 0.164		*p* = 0.334		*p* = 0.164	
a-tDCS	1.33 ± 0.90	1.67 ± 0.97	6.93 ± 0.25	6.80 ± 0.41	1.00	1.20 ± 0.41
	*p* = 0.082		*p* = 0.164		*p* = 0.055	
a-tDCS_UHCDS_	1.47 ± 0.91	1.67 ± 1.13	6.93 ± 0.25	6.80 ± 0.56	1.00	1.20 ± 0.41
	*p* = 0.082		*p* = 0.164		*p* = 0.082	


*Data showing Mean ± SD of group, scores: 1–7 (1: no attention, 7: full attention; 1:no fatigue, 7: highest fatigue level; 1: no pain, 7: maximum pain).*

### Comparison of Baseline Values

The one-way ANOVA showed that there were no significant difference between the baseline RT [*F*_(2,28)_ = 2.56, *p* > 0.05], 9-PPB [*F*(_2,28_) = 1.638, *p* > 0.05] and FMA [*F*_(2,28)_ = 0.318, *p* > 0.05] for the sham stimulation, a-tDCS, and a-tDCS_UHCDS_: (Table 3). Thus, the baseline for the sham stimulation was used as baseline measurement for all conditions in data analyses.

**Table 3:** Mean and SD of RT, 9-PPT, and FMA in three stimulation conditions.

	Sham	a-tDCS	a-tDCS_UHDCS_
RT (s)			
Pre	0.670 ± 0.048	0.604 ± 0.054	0.625 ± 0.052
Post	0.665 ± 0.048.91	0.607 ± 0.048	0.577 ± 0.037
9-PPT (s)			
Pre	70.76 ± 5.56	74.33 ± 6.40	70.23 ± 6.13
Post	70.38 ± 5.96	71.09 ± 5.80	65.75 ± 5.47
FMA			
Pre	38.20 ± 1.47	38.26 ± 1.46	38.20 ± 1.43
Post	38.46 ± 1.50	38.33 ± 1.46	38.53 ± 1.41


*RT, reaction time; 9-PPB, nine-pin pegboard; FMA, Fugl–Meyer assessment; oxa-tDCS, anodal-tDCS; oxa-tDCS_UHCDS_, anodal unihemispheric concurrent dual-site transcranial direct current stimulation. Values are mean ± SE.*

### Reaction Time

**FIGURE 3:**
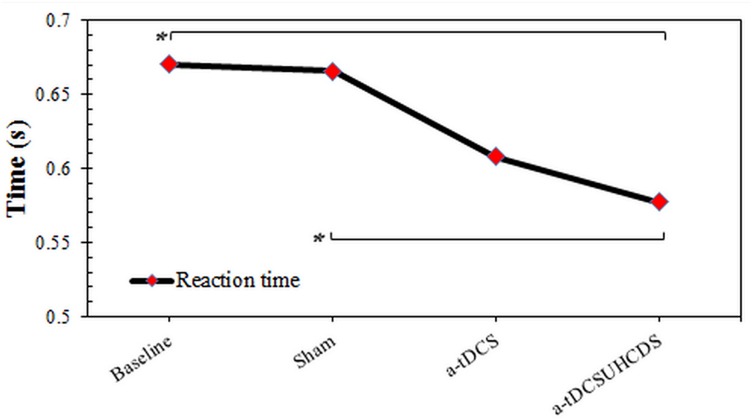
The comparison of reaction time (mean ± SD) before and after the stimulation. a-tDCS, anodal-tDCS; a-tDCS_UHCDS_, anodal unihemispheric concurrent dual-site transcranial direct current stimulation; ^∗^, significant.

The repeated measures ANOVA showed the type of stimulation had a significant effect on the RT: *F*_(2.087,29.224)_ = 4.96, *p* < 0.05 (Figure 3). The Bonferroni correction showed that after applying a-tDCS_UHCDS_, there was a significant lower RT compared with the sham stimulation (*p* = 0.031). However, there was no significant difference between a-tDCS and a-tDCS_UHCDS_ (*p* > 0.05).

### Nine-Pin Pegboard Test

**FIGURE 4:**
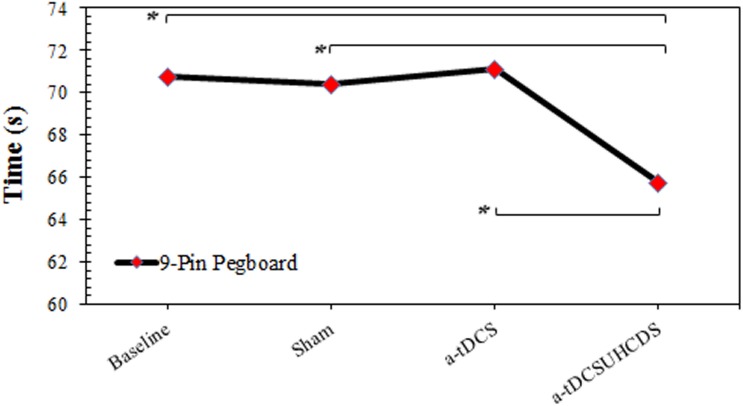
The comparison of completion time of the 9-pin pegboard test (mean ± SD) before and after the stimulation. a-tDCS, anodal-tDCS; a-tDCS_UHCDS_, anodal unihemispheric concurrent dual-site transcranial direct current stimulation; ^∗^, significant.

The repeated measures ANOVA showed the type of stimulation had a significant effect on the completion time for the 9-PPB: *F*_(3,42)_ = 5.997, *p* < 0.05 (Figure 4). There was a significant lower completion time after applying a-tDCS_UHCDS_ compared with the sham stimulation (*p* = 0.036) and a-tDCS (*p* = 0.015). However, there was no significant difference between a-tDCS and the sham stimulation (*p* > 0.05).

### Fugl–Meyer Assessment

**FIGURE 5:**
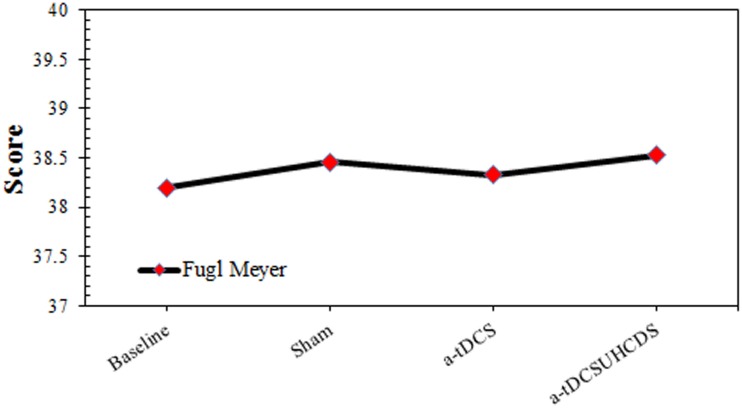
The comparison of Fugl-Meyer assessment (mean ± SD) before and after the stimulation. a-tDCS, anodal-tDCS; a-tDCS_UHCDS_, anodal unihemispheric concurrent dual-site transcranial direct current stimulation.

Comparing baseline values and post-stimulation values showed the effect of the type of stimulation on the FMA was not significant. The repeated measures ANOVA showed that the stimulation had no significant effect on the FMA scores: *F*_(3,42)_ = 2.364, *p* > 0.05 (Figure 5).

## Discussion

### Baseline Measurements

There were no significant differences between the baseline measurements of the dependent variables (i.e., RT, FMA, and 9-PPB) for the three experimental conditions. This similarity indicated that the length of the wash-out period was enough to avoid the carry-over effect between the stimulation conditions.

### Safety and Side Effects of a-tDCS_UHCDS_

Fatigue, attention, and discomfort can influence motor performance. However, there was no significant difference between the pre- and post-procedure for these scales in this study, so we concluded fatigue, attention and discomfort did not affect out the results.

### Effect of a-tDCS_UHCDS_ on Reaction Time

The results of this study indicated that a-tDCS_UHCDS_ had a more significant effect on the reduction of RT than the single-site a-tDCS and the sham stimulation. The findings supported the hypothesis that a-tDCS_UHCDS_ of the M1–DLPFC would induce a larger decrease in RT than single-site a-tDCS of the M1. This result agreed with the findings of Vaseghi et al. (2015b) who reported that the simultaneous stimulation of the M1 and DLPFC increased the CSE in healthy individuals. There is a functional connectivity between the different brain regions (Keeser et al., 2011; Luft et al., 2014). It has been shown that the premotor cortex consists of dorsal and ventral sections. The outputs of the dorsal section are sent to the M1 and spinal cord, while the premotor cortex receives inputs from the DLPFC (Dum and Strick, 1991; He et al., 1993). Therefore, we can conclude that the M1 and DLPFC are indirectly connected. Studies have also shown the functional connectivity between M1 and DLPFC (Vaseghi et al., 2015b, 2016). Therefore, compared with single-site a-tDCS of the M1, a-tDCS of the M1–DLPFC activates the DLPFC–premotor cortex–M1 pathway (Bunge et al., 2001; Nitsche and Paulus, 2001; Van Ryckeghem et al., 2013), which may have a profound effect on cortical and behavioral outcome measures, such as motor RT. In addition, there is also a connection between the ventral premotor section and the prefrontal cortex (Hutchins et al., 1988; Lu et al., 1994). Therefore, stimulating the prefrontal cortex can influence the ventral premotor section, which contains the upper limb representations (Hutchins et al., 1988; Lu et al., 1994). It should be noted that any improvement in performance, such as a reduced RT, requires high levels of cognition and improved motor performance (Salthouse, 1996; Madden, 2001). Cappon et al. (2016) in a comprehensive systematic review reported that a-tDCS stimulation of DLPFC could improve cognitive impairments in chronic stroke patients. Therefore, the simultaneous stimulation of the M1 and DLPFC not only favorably affects the CSE, but also improves the performance by decreasing the RT (Salthouse, 1996). In other words, an improved RT requires both motor and cognitive functions and the simultaneous stimulation of the M1 and DLPFC may cause motor and cognitive improvement.

Hummel et al. (2006) showed that one session of a-tDCS of M1 could significantly reduce the RT. This finding disagreed with the results of the current study because we did not observe any significant changes in the RTs of patients’ upper limbs after a-tDCS of the M1. The discrepancy between the results may be explained by the fact that different assessment tools were used. In the present study, the clinical measurements of RT were used, while Hummel et al. (2006) used electromyography to assess the RT.

### Effect of a-tDCS_UHCDS_ on Nine-Pin Pegboard Test

The results of the present study indicated that there was a significant reduction in the completion time of the 9-PPB test after a-tDCS_UHCDS_ compared with the single-site a-tDCS and the sham stimulation. This finding supported our hypothesis that a simultaneous stimulation of ipsilateral M1 and DLPFC would improve the 9-PPB completion time. This finding was consistent with the study by Vaseghi et al. (2015b), which showed an improvement in M1 excitability and motor function following the dual-site stimulation of the M1 and DLPFC. It is believed that the 9-PPB test involves a neural network for transferring the touch and visual information to the opposite M1 of the involved hand. Therefore, the 9-PPB test involves the integration of sensory, motor, and cognitive processes (Talati et al., 2005). Other studies have claimed that a-tDCS of the DLPFC improves performance in several cognitive domains, including executive functions (Keeser et al., 2011); thus, the present findings seemed reasonable. In addition, there are a number of well-known projections between the DLPFC, the cingulate cortex, and the parietal lobe. Therefore, it might be assumed that a-tDCS of the DLPFC increases the interaction between these pathways, which form part of the so-called “attention” network. Increased connectivity within this network was also reported following cognitive training and application of a-tDCS. Therefore, it is quite possible that the simultaneous stimulation of the M1 and DLPFC improves a patient’s attention, which consequently leads to a better performance in completing the 9-PPB test (Fransson, 2005; van de Ven et al., 2008; Keeser et al., 2011). In addition, Bachtiar et al. (2015) reported that a-tDCS of the M1 causes an increase in CSE. This increase coincided with a reduction in the GABAergic inhibitory system (Ziemann et al., 1998; Garry et al., 2004; Vaseghi et al., 2015b), as evidenced by a decrease in the short interval intracortical inhibition (Nitsche et al., 2005). Increased CSE in the involved cerebral hemispheres may help in the recovery of motor function of affected limbs after a stroke, which may lead to a better performance during the 9-PPB test.

### Effect of a-tDCS_UHCDS_ on Fugl–Meyer Assessment

The results of the present study showed that there was no significant difference between the scores of FMA for the different experimental conditions. This finding partly agreed with a previous studies that found there was no improvement in upper limb performance following a-tDCS of the M1 (Hesse et al., 2007; Rossi et al., 2013). In the majority of studies, a-tDCS was used as a priming technique concurrently with functional training or robotic upper limb therapy. While some of these studies reported an improvement in the FMA, no difference was observed between the a-tDCS and control groups (Kim et al., 2010; Cha et al., 2014). Hesse et al. (2011) assessed the priming effects of a-tDCS with and without robotic-assisted arm training on the motor function in subacute stroke patients. Both study groups showed an improvement in the FMA, although there was no significant difference between the two experimental conditions (Hesse et al., 2011).

Rocha et al. (2016) evaluated the effect of a-tDCS on upper limb function in chronic stroke survivors. They reported an improvement in FMA scores after a-tDCS compared with the sham stimulation. This finding did not agree with the findings in the current study. This discrepancy could be explained by the methodological differences between the studies. In the Rocha et al. (2016) study, patients received 12 a-tDCS sessions, while patients in the present study only experienced a single a-tDCS session. It may be concluded that one session of intervention was insufficient to induce an improvement in the motor function of the upper limbs (Triccas et al., 2015; Rocha et al., 2016). In addition, the sample size calculation for the present study was performed based on time-based variables such as RT and 9-PPB and not based on function-based variables like FMA. Therefore, it is possible that the small sample size of the present study resulted in insignificant finding for FMA.

### Limitations and Suggestions for Future Research

There are some limitations of the present study that may affect the interpretation of the results. First, the target population was subacute stroke patients. Therefore, it might not be possible to generalize the results to chronic stroke survivors. As it is believed that the neuroplasticity of the nervous system changes over time, the effects of a-tDCS may be different in chronic stroke patients. The second limitation is that we did not directly examine the cortex excitability. Future studies should evaluate the excitability of the cortex directly using a-tDCS_UHCDS_. Despite these limitations, this is the first study that used the novel tDCS on stroke survivors.

A future study with more a-tDCS_UHCDS_ treatment sessions, especially in combination with other rehabilitation programs, will shed more light on the rehabilitation of upper limb motor function in stroke survivors. Vines et al. (2008) suggested that the addition of cathodal tDCS (c-tDCS) on the contralateral M1 simultaneously with a-tDCS on the M1 might have a greater effect on motor function than anodal stimulation alone. This may occur because of the balancing act of the cathodal stimulation of the opposite M1, which reduces the inhibitory effects of the intact brain on the involved side (Bradnam et al., 2011). Future studies are recommended to evaluate the effects of this balancing act through the cathodal stimulation of the uninvolved hemisphere. Finally, the small sample size in the present study might limit us to give a robust conclusion especially on insignificant findings and compare and investigate effects of the site of lesion on motor improvement. Future studies with larger sample size and comparable healthy group are recommended.^1^

## Conclusion

The present study investigated the difference between the effects of conventional single-site stimulation of M1 and unihemispheric concurrent dual-site a-tDCS of the M1 and DLPFC on upper limb motor function of subacute stroke patients. We concluded that the simultaneous stimulation of the M1 and DLPFC induced a more significant reduction in RT and completion time for the 9-PPB test and, thus, caused more improvement in hand function. Therefore, a-tDCS_UHCDS_ could be used as a complementary treatment for the improvement of upper limb motor function in stroke patients.

## Data Accessibility

The data are accessible from the authors. They are not uploaded into public repositories, because the ethics agreement did not contain this.

## Author Contributions

LR, NK, IA, and SJ designed the experiments. STA collected the data. LR, STA, NK, IA, SJ, and SAA analyzed and interpreted the data and wrote the manuscript.

## Conflict of Interest Statement

The authors declare that the research was conducted in the absence of any commercial or financial relationships that could be construed as a potential conflict of interest.

## Footnotes

1 http://www.irct.ir
